# Antibiotic-induced gut dysbiosis and cognitive, emotional, and behavioral changes in rodents: a systematic review and meta-analysis

**DOI:** 10.3389/fnins.2023.1237177

**Published:** 2023-09-01

**Authors:** Shivdeep S. Hayer, Soonjo Hwang, Jonathan B. Clayton

**Affiliations:** ^1^Department of Biology, University of Nebraska at Omaha, Omaha, NE, United States; ^2^Callitrichid Research Center, University of Nebraska at Omaha, Omaha, NE, United States; ^3^Nebraska Food for Health Center, University of Nebraska-Lincoln, Lincoln, NE, United States; ^4^Department of Population Medicine, University of Guelph, Guelph, ON, Canada; ^5^Department of Psychiatry, University of Nebraska Medical Center, Omaha, NE, United States; ^6^Department of Food Science and Technology, University of Nebraska-Lincoln, Lincoln, NE, United States; ^7^Department of Pathology and Microbiology, University of Nebraska Medical Center, Omaha, NE, United States; ^8^Primate Microbiome Project, University of Nebraska-Lincoln, Lincoln, NE, United States

**Keywords:** gut microbiota, behavior, anxiety, depression, social, antibiotics, anhedonia, microbiota-gut-brain axis

## Abstract

There are previous epidemiological studies reporting associations between antibiotic use and psychiatric symptoms. Antibiotic-induced gut dysbiosis and alteration of microbiota-gut-brain axis communication has been proposed to play a role in this association. In this systematic review and meta-analysis, we reviewed published articles that have presented results on changes in cognition, emotion, and behavior in rodents (rats and mice) after antibiotic-induced gut dysbiosis. We searched three databases—PubMed, Web of Science, and SCOPUS to identify such articles using dedicated search strings and extracted data from 48 articles. Increase in anxiety and depression-like behavior was reported in 32.7 and 40.7 percent of the study-populations, respectively. Decrease in sociability, social novelty preference, recognition memory and spatial cognition was found in 18.1, 35.3, 26.1, and 62.5 percent of the study-populations, respectively. Only one bacterial taxon (increase in gut *Proteobacteria*) showed statistically significant association with behavioral changes (increase in anxiety). There were no consistent findings with statistical significance for the potential biomarkers [Brain-derived neurotrophic factor (BDNF) expression in the hippocampus, serum corticosterone and circulating IL-6 and IL-1β levels]. Results of the meta-analysis revealed a significant association between symptoms of negative valence system (including anxiety and depression) and cognitive system (decreased spatial cognition) with antibiotic intake (*p* < 0.05). However, between-study heterogeneity and publication bias were statistically significant (*p* < 0.05). Risk of bias was evaluated to be high in the majority of the studies. We identified and discussed several reasons that could contribute to the heterogeneity between the results of the studies examined. The results of the meta-analysis provide promising evidence that there is indeed an association between antibiotic-induced gut dysbiosis and psychopathologies. However, inconsistencies in the implemented methodologies make generalizing these results difficult. Gut microbiota depletion using antibiotics may be a useful strategy to evaluate if and how gut microbes influence cognition, emotion, and behavior, but the heterogeneity in methodologies used precludes any definitive interpretations for a translational impact on clinical practice.

## Introduction

Antibiotics are “miracle” drugs that have saved the lives of countless humans suffering from infectious diseases. However, the side-effects of these drugs are not fully known. In particular, the relationship between antibiotic intake and onset of neuro-psychiatric symptoms/syndromes such as depression and anxiety has been postulated based on large scale epidemiological studies in humans. An association between antibiotic use and higher risk for depression and anxiety was found in a nested case–control study in the United Kingdom (Lurie et al., 2015). A study showed a correlation between the high concentrations of antibiotics in urine and presence of depression in a cohort of elderly people in China (Liu et al., 2021). Mental health problems such as peer problems, hyperactivity and conduct issues were also associated with ciprofloxacin use in children in China (Zhang J. et al., 2021).

The gut microbiome is comprised of all the microorganisms in the gut including bacteria, viruses, and fungi and the bi-directional communication between the gut and brain mediated via actions of bacteria is referred to as the “microbiota-gut-brain axis” (Olavarría-Ramírez et al., 2023). Gut dysbiosis has been implicated in the pathogenesis of numerous human diseases including autoimmune diseases, metabolic disorders, cancer, and pertaining to our study, psychiatric disorders (Duvallet et al., 2017; Sherwin et al., 2019; Simpson et al., 2020). On the other hand, microbes (particularly probiotics) have also been studied for their beneficial effects in reducing stress and anxiety (Amirani et al., 2020; Zhang et al., 2020; Ma et al., 2021).

Antibiotics could potentially create the most potent disruption of the microbiota-gut-brain axis, which has significant implications in both basic and translational research (Ramirez et al., 2020). The mechanistic links between antibiotic-mediated gut dysbiosis and induction of cognitive, emotional, and behavioral changes have been studied in numerous rodent studies and recently reviewed (Hao et al., 2020). Briefly, these mechanisms include changes in circulating inflammatory cytokine levels, oxidative stress, inhibition of brain-derived neurotrophic factor (BDNF) expression, decrease in neurotransmitter concentrations in brain tissues and blood, changes in short chain fatty acid levels in gut, and disruption of vagal nerve activity (Hao et al., 2020).

There has been a spurt of articles linking antibiotics with cognitive, emotional, and behavioral changes in rodents in recent years. However, the results reported in these studies lack consensus and are often contradictory (Olavarría-Ramírez et al., 2023). In this systematic review and meta-analysis, we critically reviewed all the published articles that have presented results on changes in cognition, emotion, and behavior in rodents (rats and mice) after antibiotic-induced gut dysbiosis.

## Methods

### Narrative systematic review

A dedicated search string was created for three different databases (Web of Science, PubMed, and Scopus; Supplementary material 1). These search strings consisted of words that describe the four major items of this review: antibiotics, rodents, behaviors, and gut microbiome. The inclusion criteria included studies that contrasted changes in behavior between antibiotic-treated and vehicle-treated groups and described changes in gut microbiome in rats and mice. The articles were excluded if:

1. Either of the above items were missing from the article.
2. Strains of rodents used were created to model a specific disease (e.g., NOD mice for diabetes, *15qdup* for autism spectrum disorder).
3. There were other confounding factors such as probiotic administration, presence of a stressor, fecal microbiota transplantation, etc.
4. Behaviors other than those of interest (Table 1) were evaluated.
5. Articles were written in a language other than English.

**Table 1:** Brief description of the behavioral tests included in this review.

Behavioral test	Brief synopsis	Behavioral assessment	Behavioral phenotype
Open field test (OFT)	Anxious mice are less likely to explore open spaces and will stick closer to walls	Time spent in center	Anxiety
Elevated plus maze test (EPM)	Anxious rodents are less likely to explore the open arms of an elevated platform	Time spent in open	Anxiety
Light/Dark box test (L/D box)	Anxious rodents are less likely to explore the illuminated chamber	Time spent in light	Anxiety
Morris water maze test (MWM)	After training for a few days, rodents would remember the quadrant where the platform is located in the water cylinder	Time in target quadrant	Spatial cognition
Morris water maze test (MWM)	Time taken for rodents to identify hidden platform in water and escape	Escape latency	Spatial cognition
Novel object recognition test (NOR)	Rodents tend to interact more with a novel object	Discrimination index	Recognition memory
Forced swimming test (FST)	Depressed rodents spend less time swimming as a coping mechanism	Immobility time	Depression
Tail suspension test (TST)	Depressed rodents spend less time struggling to escape when suspended from tail	Immobility time	Depression
Three-chambered sociability test (3-SC)	Rodents spend more time with a rodent than an object	Time spend interacting with rodent	Sociability
Three-chambered sociability test (3-SC)	Rodents spend more time with a new rodent as compared to a familiar rodent	Time spend interacting with new rodent	Social novelty
Sucrose preference test (SPT)	Rodents prefer sugary water over unflavored water	Ratio of sugar water intake and total water intake	Anhedonia

**Figure 1:**
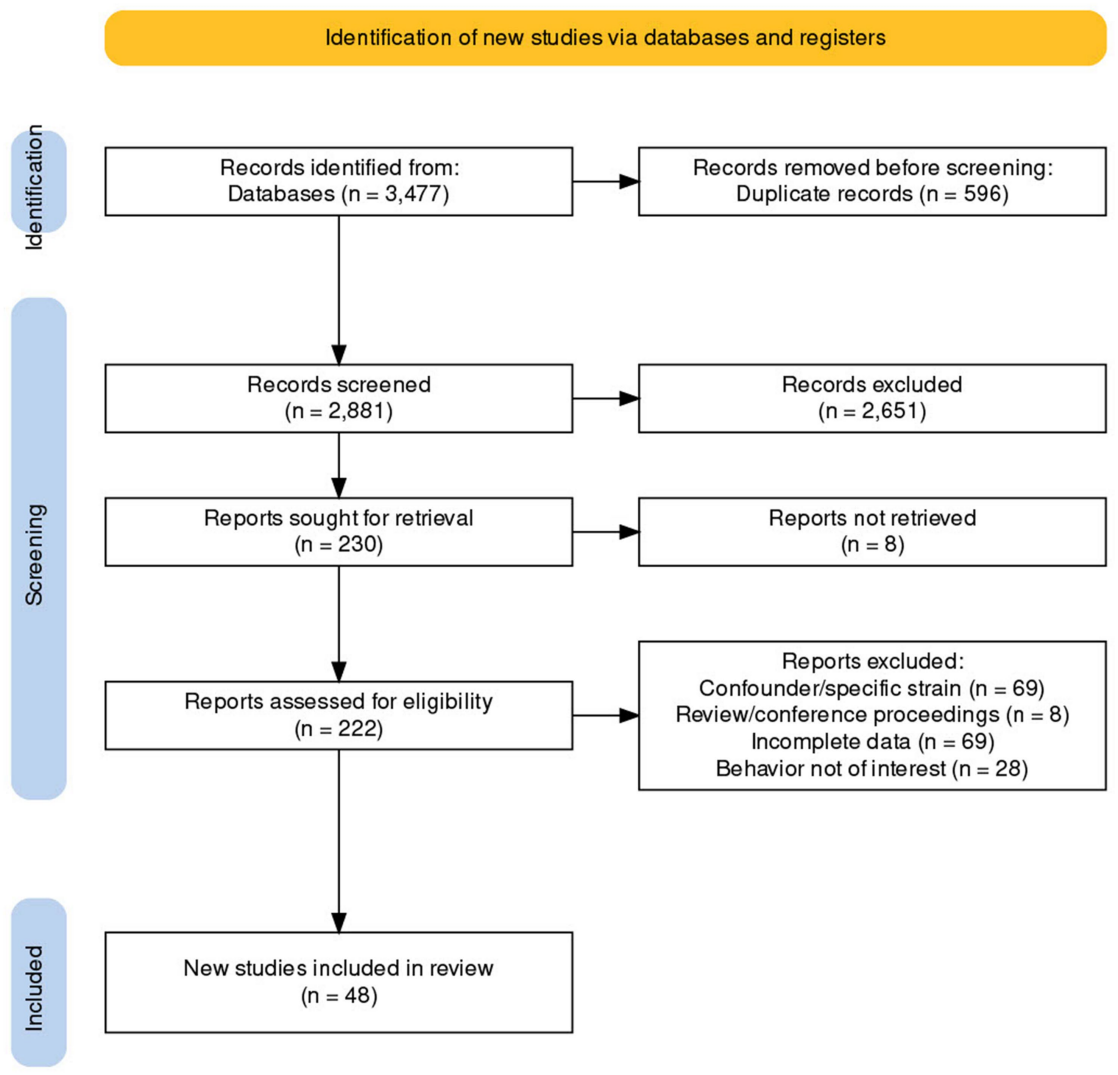
PRISMA flow diagram of article selection for this systematic review.

The search was initially conducted on January 3, 2022 and revised on January 20, 2023. The details of the literature search conducted are presented in Figure 1. We extracted the following data:

- Name of the last author and title of the publication.
- Methodology to study gut bacterial population, bacterial taxa (phyla and genera level).
- Details of the behavioral outcomes.
- Molecular mechanisms potentially associated with changes in behavior after antibiotic-induced gut dysbiosis (BDNF in brain tissues, cytokines in blood and brain, and serum corticosterone).
- Antibiotics used, route and duration of antibiotic administration, time between antibiotic administration, and behavioral assessment.
- Rodent species, strain, sex, and age.

Some studies described results of experiments conducted on multiple populations such as those differentiated by mice strains, sexes, age-groups or antibiotics used within the same study. Hence, for the purpose of this review, the unit of analysis is the “population” within an article.

The tests included in this review are open field test (OFT), elevated plus maze test (EPM), light–dark box test (L/D), forced swim test (FST), tail suspension test (TST), Morris water maze test (MWM), novel object recognition test (NOR), three-chambered sociability test (sociability and social novelty), and sucrose preference test (SPT). We have provided a brief description of the tests (Table 1) and their relevance to Research Domain Criteria (Morris and Cuthbert, 2012) in Table 2.

**Table 2:** Interpretation of the behavioral tests in the context of research domain criteria.

Behavioral test	Behavioral domain in research domain criteria
Open field test	Negative valence system: potential threat
	Positive valence system: reward responsiveness – reward anticipation
	Sensorimotor system: motor actions – initiation, execution, and inhibition/termination
Elevated plus maze test	Negative valence system: potential threat
	Positive valence system: reward responsiveness – reward anticipation
	Sensorimotor system: motor actions – initiation, execution, and inhibition/termination
Light/Dark box test	Negative valence system: acute threat and potential threat
Morris water maze test	Cognitive system: working memory – active maintenance
	Cognitive system: working memory – flexible updating
	Cognitive system: working memory – interference control
	Sensorimotor system: motor actions – execution
Novel object recognition test	Cognitive system: working memory – active maintenance
	Cognitive system: working memory – flexible updating
	Cognitive system: working memory – interference control
	Sensorimotor system: motor actions – execution
Forced swimming test	Negative valence system: acute threat
	Negative valence system: sustained threat
	Negative valence system: frustrative nonreward
	Sensorimotor system: motor actions – initiation
	Sensorimotor system: motor actions − execution
Tail suspension test	Negative valence system: acute threat
	Negative valence system: sustained threat
	Negative valence system: frustrative nonreward
	Sensorimotor system: motor actions – initiation
	Sensorimotor system: motor actions − execution
Three-chambered sociability test	Systems for social processes: affiliation and attachment
	Systems for social processes: social communication
	Systems for social processes: perception and understanding of others
Sucrose preference test	Positive valence system: reward anticipation
	Positive valence system: initial response to reward
	Positive valence system: reward satiation
	Positive valence system: reward valuation – effort

We generated a behavioral phenotype (e.g., decrease in anxiety) after assessing each behavioral outcome carefully for each study population. Some of the studies utilized multiple tests to study the same phenotype. For example, OFT, EPM, and L/D box test the unconditioned, exploratory behavior of the rodents, which is used to assess “anxiety-like” behavior. If a population was evaluated for the same phenotype using multiple tests, then such population was considered to be positive for said phenotype even if only one of the tests were positive.

Risk of bias assessment was conducted by modifying the SYRCLE’s risk of bias tool (Hooijmans et al., 2014). The following items were evaluated for this assessment: randomization, baseline characteristics, blinding, husbandry characteristics, attrition bias, selective outcome reporting of behavioral assessment, and microbiome analysis.

### Meta-analysis

Summary statistics of the behavioral assessments were usually not provided in the articles selected. We extracted the mean and variance estimates (standard deviation and standard error) from the figures using WebPlotDigitizer version 4.6 online (Rohatgi, 2014). Data was extracted for the following behavioral outcomes: time spent in center (OFT), immobility time (FST), immobility time (TST), time spent in light (L/D box), discrimination index (NOR), time spent in open arm (EPM), time in target quadrant (MWM), and time to latency (MWM). Since different studies used different time scales, we first estimated standardized mean differences. Hierarchical random effects models were built for each behavioral outcome separately to estimate pooled standardized mean differences using the package “metafor” (version 2.4–0) in R version 2022.12 (Viechtbauer, 2010). The model specifications included selecting t-distribution to model test statistics, restricted maximum likelihood estimation for determining the variance and the article as a random effect. Two types of models were built for each behavioral outcome separately: Model A consisting of all the available data and Model B consisting of data from male mice administered antibiotics orally. The latter was chosen as this was by far the most common population characteristic in the studies selected. These models also provided a test of heterogeneity as part of the default output.

Subgroup analysis was conducted to evaluate the impact of age at which antibiotic administration was initiated (pre-natal and early post-natal vs. adolescent-adult), duration of antibiotic treatment (less than 14  days vs. more than 14  days), number of antibiotics administered (single vs. multiple), and time between antibiotic treatment and behavioral assessment (immediate vs. several days). Publication bias was assessed using Egger’s test (Egger et al., 1997).

Formal meta-analysis of microbiome data was not conducted because the data were not extractable from the graphs reported in the articles and raw reads were often not available in the public repositories. Hence, we conducted a semi-quantitative analysis to check if the direction of significant changes in gut microbiome (increase, decrease, or no change) were associated with behavioral changes (increase, decrease, or no change) using Fisher’s exact test (2 × 2 tables) or Freeman–Halton extension of Fischer’s exact test (3 × 3 or 3 × 2 tables). A meta-analysis of molecular mechanisms was not conducted because these were secondary outcomes and were not part of the search strings.

## Results

### Description of the articles

The details of the literature search conducted are presented in Figure 1. The complete data extracted are available in Supplementary material 2. We eventually included 48 articles in this review. Seventy-three different “populations” of animals were experimented on in these 48 studies. Thirty-three studies had data on only one population and 15 studies had data on multiple populations.

Mice and rats made up the subjects in 66 (90.4%) and 7 (9.6%) of these populations, respectively. C57BL/6 J was the most common strain of mice used (34 populations), followed by BALB/C (13 populations). Cocktails consisting of several antibiotics were tested on 42 (57.5%) populations and the rest of the populations (*n* = 31, 42.5%) were tested with a single antibiotic. Oral route of antibiotic administration was by far the most common route of antibiotic administration (69 populations, 94.5%). Fifty-seven (78.1%) of these populations were comprised of male rodents only, and 10 (13.7%) and six (8.2%) populations were made up of female rodents only and rodents of both sexes, respectively. Antibiotics were administered on an average for 22.4 days (range: 1–150 days). The average time between the last day of antibiotic administration and start of behavioral testing was 14.3 days (range: 0–98 days). None of the articles reported an increase in anhedonia after antibiotic administration (based on SPT; Wang et al., 2020a,b; Li et al., 2021; Hassan et al., 2022; Yao et al., 2022; Yu et al., 2022). Anhedonia will not be discussed further in the subsequent paragraphs.

### Risk of bias

The average risk of bias score was 3.67 (range: 2–7) out of a possible highest score of 7. Behavioral outcomes and husbandry descriptions (including environmental conditions, housing, and feeding) were adequately reported in 48 (100%) and 46 (95.8%) articles, respectively. Randomization, microbiome data availability, baseline characteristics, blinding, and attrition bias were explained in only 54.2, 43.8, 12.5, 29.2, and 31.3% of the articles, respectively. Fecal and cecal contents were sampled in 32 (66.7%) and 16 (33.3%) of the articles, respectively. Storage temperatures or time between sample collection and DNA extraction of fecal/cecal contents was not provided in 25 (52.1%) articles. When provided, fecal samples were stored at −20 or −80^o^C without any additives.

### General results of the studies

Decrease in sociability and social novelty preference was reported in 18.1% (4/22) and 35.3% (6/17) populations, respectively. Increase in anxiety was reported in 32.7% (19/58) populations. Increase in depression-like behavior was reported in 40.7% (11/27) populations. Decreased recognition memory and spatial cognition was observed in 26.1% (6/23) and 62.5% (10/16) populations based on NOR and MWM tests, respectively. Interestingly, a decrease in anxiety was also reported in 17.2% (10/58) of the populations.

### Changes in behavior in mice at different life stages

#### Early postnatal phase

Antibiotics were administered to mice in the early postnatal phase of life in seven articles (16 populations; Table 3). Kayyal et al. (2020) reported that oral penicillin administration led to a decrease in sociability but not anxiety in male mice without any behavioral changes in female mice 21 days post-antibiotic intake. Orally administered ampicillin and vancomycin alone caused significant increase in anxiety and decrease in social novelty, spatial cognition and sociability (except in ampicillin-treated mice) 14 days after cessation of treatments (Liu et al., 2022).

**Table 3:** Changes in behaviors in rodents in early postnatal stage of life after antibiotic administration.

Article	Strain	Sex	Antibiotics	Duration of treatment	Time between treatment and behavioral testing	Sociability	Social novelty	Spatial cognition	Recognition memory	Depression	Anxiety
Desbonnet et al. (2015)	NIH Swiss	M	Ampicillin, vancomycin, neomycin, and metronidazole	24 days	None				Decrease		Decrease
Keogh et al. (2021)	C57BL/6 J	B	Ampicillin, neomycin, and vancomycin	16 days	28 days				Decrease		Decrease
Kayyal et al. (2020)	BALB/C	B	Penicillin V	7 days	21 days	±					NS
Li et al. (2022)	KM	M	Ampicillin, vancomycin, neomycin, bacitracin, and imipenem	21–84 days	0–63 days			±		±	Increase
Zhang et al. (2022)	KM	M	Ampicillin, vancomycin, neomycin, bacitracin, and imipenem	75 days	None			Decrease		Increase	NS
Lynch et al. (2023)	NIH Swiss	B	Ampicillin, gentamicin, vancomycin, and imipenem	7 days	15–35 days	NS	NS		NS	NS	±
Liu et al. (2022)	C57BL/6 J	B	Ampicillin or vancomycin	28 days	14 days	±	Decrease	Decrease			Increase

“M,” male; “B,” both sexes; NS, no change in behavior; ±, different changes in behavior across populations within a study.

Li et al. (2022), Zhang et al. (2022), Desbonnet et al. (2015), and Keogh et al. (2021) observed decreased spatial cognition and recognition memory after oral intake of solution containing ampicillin, vancomycin, and other antibiotics. However, Li et al. (2022) and Zhang et al. (2022) found an increase, and Desbonnet et al. (2015) and Keogh et al. (2021) found a decrease in anxiety in these mice. Finally, Lynch et al. (2023) delivered a cocktail containing ampicillin, vancomycin, and other antibiotics to mice of both sexes and did not observe changes in sociability, social novelty, recognition memory, depressive-signs, and anxiety (with the exception of two of the six populations evaluated in this study).

#### Adolescent phase

Antibiotics were administered to mice in the adolescent phase of life in 23 articles (30 populations; Table 4). None and only one of the study populations displayed changes in sociability and social novelty, respectively after antibiotic administration regardless of sex, strain, or duration between treatments and behavioral observations (Table 4; Gacias et al., 2016; Guida et al., 2018; Lach et al., 2020; Zhang Z. et al., 2021). Similarly, recognition memory decreased in only one of the study populations tested (Guida et al., 2018; Lach et al., 2020; Saunders et al., 2020; Arslanova et al., 2021; Luo et al., 2022). Recognition memory was decreased in male mice administered a cocktail of four antibiotics intraperitoneally for 14 days and immediately followed by behavioral testing (Arslanova et al., 2021). Spatial cognition decreased in only two of the five study populations (Wang et al., 2021; Zheng et al., 2021; Li et al., 2022). These two study populations consisted of C57BL/6 J administered vancomycin for 77 days (Zheng et al., 2021) and ampicillin, streptomycin, and clindamycin for 21 days (Wang et al., 2021).

**Table 4:** Changes in behaviors in rodents in adolescent stage of life after antibiotic administration.

Article	Strain	Sex	Antibiotics	Rx duration	Time between rx and behavioral testing	Sociability	Social novelty	Spatial cognition	Recognition memory	Depression	Anxiety
Septyaningtrias et al. (2020)	C57BL/6 J	M	amp or neo	28	None						NS
Hassan et al. (2022)	C57BL/6 J	M	neo, van, and mer	28	None						Decrease
Reyes et al. (2020)	C57BL/6 J	M	bac, neo, van, and pim	14	None						NS
Guida et al. (2018)	C57BL/6 J	M	amp, str, and cli	14	14	NS	Decrease		NS	Increase	
Wang et al. (2021)	C57BL/6 J	M	amp, str, and cli	21	None			Decrease		Increase	Increase
Zheng et al. (2021)	C57BL/6 J	M	van	21–77	None			±			
Lach et al. (2020)	C57BL/6 J	M	amp, van, cip, imi, and met	21	24	NS	NS		NS		Increase
Jang et al. (2018)	C57BL/6 J	M	amp	2	10						Increase
Saunders et al. (2020)	CD1	M	str	1	77				NS		
Wang et al. (2020a)	C57BL/6 J	M	amp, neo, and met	14	14					NS	
Zhao et al. (2020)	BALB/C	M	axo	77	None					Increase	Increase
Grant et al. (2021)	C57BL/6 J	F	neo, van, and met	8	None						Increase
Gacias et al. (2016)	C57BL/6 J	M	neo, van, met, and amp (SC, oral)	14	None	NS				NS	NS
Arslanova et al. (2021)	Unknown	M	amp, van, neo, and met (IP)	14	None				Decrease		Increase
Ray et al. (2021)	C57BL/6 J or BALB/C	M	van	6	None-54					±	±
Hao et al. (2021)	C57BL/6 J	M	amp	10	None					Increase	Increase
Zhang Z. et al. (2021)	KM	F	neo, van	21	21	NS	NS				
Lai et al. (2022)	C57BL/6 J	M	amp, bac, neo, and van	14	None						NS
Li et al. (2022)	KM	M	amp, van, neo, bac, and imi	28	None			NS		NS	NS
Luo et al. (2022)	C57BL/6 J	M	amp, van, neo, gen, and ery	28	28				NS		
Tang et al. (2022)	BALB/C	B	azi	63	7						Increase
Sun et al. (2023)	C57BL/6 J	M	amp, str, and cli	21	14					Increase	Increase

“M,” male; “B,” both sexes; “F,” female; NS, no change in behavior; ±, different changes in behavior across populations within a study; amp, ampicillin; neo, neomycin; van, vancomycin; mer, meropenem; bac, bacitracin; pim, pimaricin; str, streptomycin; cli, clindamycin; cip, ciprofloxacin; imi, imipenem; met, metronidazole; axo, ceftriaxone; gen, gentamicin; ery, erythromycin; azi, azithromycin rx-treatment. Durations and times were measured in days.

Unlike the results described above, antibiotics were able to induce depression-like behavior-and anxiety with more consistency. Oral intake of ampicillin or ceftriaxone alone was sufficient to increase depression-like behavior after 10 and 77 days of intake, respectively (Zhao et al., 2020; Hao et al., 2021). Ray et al. (2021) reported an increase in depression-like behavior in C57Bl/6 J mice after oral vancomycin administration for 6 days, but not in BALB/C mice. A combination of ampicillin, streptomycin, and clindamycin administered orally for 14–21 days led to an increase in depression-like behavior even after 14 days of last treatment (Guida et al., 2018; Wang et al., 2021; Sun et al., 2023). Interestingly, administration of combination of ampicillin, neomycin and/or vancomycin did not lead to changes in depression-like behavior regardless of strain, route of treatment, treatment duration, and duration between treatments and behavioral observations (Gacias et al., 2016; Wang et al., 2020b; Li et al., 2022).

Oral intake of ampicillin, ceftriaxone, vancomycin, or azithromycin alone was sufficient to induce anxiety (Jang et al., 2018; Zhao et al., 2020; Hao et al., 2021; Ray et al., 2021; Tang et al., 2022). However, in one experiment, oral ampicillin or neomycin alone did not induce anxiety in mice (Septyaningtrias et al., 2020). A cocktail of ampicillin, streptomycin, and clindamycin administered orally led to increased anxiety in two separate experiments (Wang et al., 2021; Sun et al., 2023). Impact of the oral administration of other antibiotic combinations on the anxiety of mice was ambiguous, with an increase and decrease in anxiety reported in 2 and 1 of the seven articles, respectively (Table 4). Finally, intraperitoneal administration of ampicillin, vancomycin, metronidazole, and neomycin for 14 days led to an increase in anxiety (Arslanova et al., 2021) but not when the same cocktail was administered subcutaneously for the same duration (Gacias et al., 2016).

#### Adult phase

Ten studies evaluated behavioral changes after antibiotic administration in 13 populations of adult mice (Table 5). Oral administration of ampicillin alone for 14 days was sufficient to cause decrease in spatial cognition, recognition memory, and anxiety (Roy Sarkar et al., 2020; Sarkar et al., 2022). Morozova et al. (2022) reported decrease in anxiety after oral vancomycin and rifampin administration. Fröhlich et al. (2016) found a decrease in recognition memory without changes in depressive-signs and anxiety after oral administration of an antibiotic cocktail for 7 days. Mezö et al. (2020) found no changes in recognition memory even after administering a cocktail of antibiotics for 60 days.

**Table 5:** Changes in behaviors in rodents in adult stage of life after antibiotic administration.

Article	Strain	Sex	Antibiotics	Duration of treatment	Time between treatment and behavioral testing	Sociability	Social novelty	Spatial cognition	Recognition memory	Depression	Anxiety
Luo et al. (2021)	C57BL/6 J	M	Cefazolin (IP)	5 days	3 days			NS			
Fröhlich et al. (2016)	C57BL/6 J	M	Ampicillin, bacitracin, meropenem, neomycin, vancomycin	7 days	None				Decrease	NS	NS
Mezö et al. (2020)	C57BL/6 J	M	Vancomycin, Cefoxitin, Gentamicin, Metronidazole	60 days	None				NS		
Lach et al. (2020)	C57BL/6 J	M	Ampicillin, Vancomycin, Ciprofloxacin, Imipenem, Metronidazole	21 days	24 days	NS	NS		NS		NS
Geary et al. (2021)	C57BL/6 J	B	Neomycin, Bacitracin, Pimaricin	5 days	None						±
Bercik et al. (2011)	BALB/C	M	Neomycin, Bacitracin, Pimaricin (oral, IP)	7 days	None-14 days						±
Sarkar et al. (2022)	Swiss Albino	M	Ampicillin	14 days	None			Decrease	Decrease		Decrease
Roy Sarkar et al. (2020)	Swiss Albino	M	Ampicillin	14 days	7 days			Decrease	Decrease		Decrease
Morozova et al. (2022)	C57BL/6 J	M	Vancomycin, rifampicin	14 days	None						Decrease
Yao et al. (2022)	C57BL/6 J	M	Minocycline	14 days	None					NS	NS

“M,” male; “B,” both sexes; NS, no change in behavior; ±, different changes in behavior across populations within a study.

Geary et al. (2021) and Bercik et al. (2011) used an identical oral antibiotic cocktail for varying durations to evaluate changes in anxiety with mixed results. Geary et al. (2021) reported a decrease in anxiety in males but not in females. A decrease in anxiety was observed in male mice evaluated for behavior immediately after cessation of oral antibiotic intake, but not if behavior was evaluated 2 weeks after last dose of oral antibiotics or if the antibiotics were administered intraperitoneally (Bercik et al., 2011; Geary et al., 2021). Finally, no changes in behavior was observed by Yao et al. (2022), Lach et al. (2020), or Luo et al. (2021) after oral minocycline, oral antibiotic cocktail and intraperitoneal administration of cefazolin, respectively (Lach et al., 2020; Luo et al., 2021; Yao et al., 2022; Table 5).

### Changes in behavior in offspring born to pregnant mice administered antibiotics

We identified five studies which evaluated the impact of microbiome changes in pregnant mice administered antibiotics on the behavior of offspring hence born. No changes in recognition memory were reported in newborn male offspring born to mothers given ampicillin, metronidazole, vancomycin, and neomycin (Bello-Medina et al., 2022). Studies also reported behavioral changes in offspring born to mothers given antibiotic cocktails but the results were inconsistent and cannot be generalized. A decrease in social novelty preference without change in sociability was observed in 98 days-old mice of either sex born to mothers given neomycin and vancomycin (Zhang Z. et al., 2021). Tochitani et al. (2016) reported an increase in anxiety without changes in sociability in 28 day-old mice of either sex born to mothers administered neomycin, bacitracin, and pimaricin.

Leclercq et al. (2017) and Champagne-Jorgensen et al. (2020) used nearly identical experimental protocols but found conflicting results. Pregnant BALB/C mice were fed low-dose penicillin V and behavior was evaluated in 42 day-old male and female offspring. Leclercq et al. (2017) reported decreased sociability and social novelty preference in male and female offspring but these changes were not reported by Champagne-Jorgensen et al. (2020). Both of these studies reported no changes in anxiety. However, Leclercq et al. (2017) and Champagne-Jorgensen et al. (2020) measured a decrease in anxiety in male offspring and female offspring, respectively. This might also indicate the presence of differences in behavioral responses to gut-brain axis perturbations based on sex.

### Changes in behavior in rats

Six studies evaluated changes in behavior in male rats after antibiotic administration. Hoban et al. (2016) and Ruiz-González et al. (2022) administered a cocktail of multiple antibiotics to 8–10 weeks old rats for 4–7 weeks and reported an increase in depression. Hoban et al. (2016) also reported decreased spatial cognition in these subjects but found no changes in anxiety and recognition memory. Cho et al. (2020), Leigh et al. (2020), Li et al. (2021), and Yu et al. (2022) administered a single antibiotic (penicillin G, minocycline, rifaximin, and imipenem, respectively) to rats and reported no changes in anxiety, anhedonia, spatial cognition, and recognition memory (not all of these behaviors were tested in each study). The context of these studies ranged from administering antibiotics to pregnant females and evaluating behavior in offspring of either sex (Cho et al., 2020), to adolescent (Li et al., 2021), and adult rats (Leigh et al., 2020; Yu et al., 2022).

### Associations between gut microbiome and behavioral changes

Overall, 180 bacterial taxa were reported in the articles (Supplementary material 2). Not all the taxa were reported in every article. These included 22 bacterial phyla and 158 genera. Of these, 12 phyla and 55 genera were not correlated with any behavioral changes. *Lactobacillus*, *Bacteroides*, *Escherichia*, *Parabacteroides*, and *Odoribacter* were the top five most reported bacterial genera correlated with changes in behavior; and changes in these genera were associated with changes in 23, 18, 14, 13, and 13 behaviors across the different study populations, respectively (Table 6). Similarly, *Firmicutes*, *Proteobacteria*, *Bacteroidetes*, *Actinobacteria*, and *Verrucomicrobia* were the most reported bacterial phyla (Table 6). The details for other taxa-behavior correlations are available in Supplementary material 2.

**Table 6:** Most commonly bacterial taxa reported in the studies included in the review and the direction of changes in the relative abundance of these taxa relative to direction of changes in rodent behavior.

Taxa	↑ Anxiety	↑ Depression	↓ Recognition memory	↓ Spatial cognition	↓ Social novelty	↓ Sociability
Phylum						
*Firmicutes*	↑3, ↓10	↓7	↓3, ↑1	↓7	↓4, ↑2	↓1, ↑3
*Proteobacteria*	↑9, ↓4	↑6	↑3	↑5	↑6	↑3
*Bacteroidetes*	↑6, ↓2	↓4	↓2	↓2	↓4	↓2
*Actinobacteria*	↑1, ↓3	↑1, ↓1	↓1	-	↑1	-
*Verrucomicrobia*	↑2, ↓1	↑1	↑1	↑1	-	-
Genus						
*Lactobacillus*	↑4, ↓6	↓4	↓3, ↑1	↓4, ↑1	↓1	-
*Bacteroides*	↑8, ↓1	↑1, ↓1	↓1	↑3	↑2	↑1
*Escherichia/Shigella*	↑7, ↓2	↑2, ↓1	-	↓1, ↑1	-	-
*Parabacteroides*	↑3, ↓4	↑1	-	↑2	↑2	↑1
*Odoribacter*	↑2, ↓4	-	↓1	↓2	↓3	↓1

↑, increase; ↓, decrease. For example, increase in anxiety was associated with decrease in Firmicutes in 10 populations.

Notably, the data at a behavior-taxa combination were very scant. However, some patterns were still evident. For example, levels of gut *Firmicutes* emerged as a “positive” indicator of behavioral status, and a decrease in *Firmicutes* was correlated with an increase in anxiety, depression-like behavior and decrease in spatial cognition (Table 6). Conversely, increase in *Proteobacteria* correlated with increased anxiety, depression-like behavior and decreased interest in novel mice and spatial cognition (Table 6). At a bacterial genera level, increase in gut *Lactobacillus* and *Bacteroides* levels were associated with increase in anxiety (Table 6). However, results of such associations were often conflicting at a descriptive level (Table 6). There was a statistically significant association between increase in gut *Proteobacteria* and increase in anxiety in rodents (Fischer’s exact test, *p* < 0.05). However, the data was usually too scant to evaluate associations between bacterial taxa and changes in behavior.

The changes in gut microbiome after administration of the same antibiotic or antibiotic combinations were not consistent across studies. Hao et al. (2021) and Sarkar et al. (2022) administered ampicillin to mice for 10 days, but only the latter reported a decrease in *Lactobacillus* spp. However, these studies differed in the dosage of ampicillin used. Gut *Firmicutes* levels increased after penicillin V administration only in 14 days old male mice, but not in females (Kayyal et al., 2020). Sex-related differences in response to the same antibiotics were also reported by Lynch et al. (2023). Gut microbiome changes in response to neomycin and recovery of gut microbiome also differed by the strain of the mice used (Ray et al., 2021). Lach et al. (2020) observed drastically different changes in gut microbiome after oral administration of antibiotics in adolescent and adult mice. Number of days between antibiotic intake and evaluation of gut microbiome (Bercik et al., 2011), and route of administration were other notable factors that could influence the gut bacterial populations (Bercik et al., 2011; Gacias et al., 2016).

### Changes in molecular mechanisms

Brain-derived neurotrophic factor (BDNF) gene expression in the hippocampus, circulating levels of IL-6 and IL-1β and serum corticosterone were the three most common molecular mechanisms studied (22, 14, 13, and 13 populations, respectively; Table 7). Results of other molecular mechanisms are presented in the Supplementary material 2. Similar to the results described in the preceding sections, changes in the expression or concentrations of these molecules were not consistent. BDNF gene expression in the hippocampus decreased in 27.3% but did not change in 68.2% of the study populations after antibiotic administration (Table 7). Post antibiotic administration, circulating levels of IL-6 and IL-1β did not change in 71.4 and 76.9 percent of the study populations, respectively (Table 7). Serum corticosterone levels increased in 38.5% but did not change in 46.2% of the study populations after antibiotic administration, respectively. Other factors such as age, sex, duration of antibiotic treatment, and time between last antibiotic and sample collection might have influenced the changes in these molecular mechanisms, but the results were not generalizable.

**Table 7:** Changes in key molecular biomarkers of behavioral changes (BDNF), endocrine signaling (corticosterone), and systemic inflammation (interleukins) after antibiotic administration.

Molecular mechanism	Direction of change	Number of populations	Article
IL-6 (circulating)	Increase	3	Leclercq et al. (2017); Jang et al. (2018); Zhao et al. (2020)
	Decrease	1	Leclercq et al. (2017)
	No change	10	Fröhlich et al. (2016); Kayyal et al. (2020); Wang et al. (2020a,b); Li et al. (2022); Zhang et al. (2022)
IL-1β (circulating)	Increase	2	Jang et al. (2018); Hao et al. (2021)
	Decrease	1	Li et al. (2022)
	No change	10	Fröhlich et al. (2016); Leclercq et al. (2017); Guida et al. (2018); Kayyal et al. (2020); Zhang et al. (2022)
BDNF expression (hippocampus)	Increase	1	Bercik et al. (2011)
	Decrease	6	Desbonnet et al. (2015); Guida et al. (2018); Jang et al. (2018); Kayyal et al. (2020); Ray et al. (2021)
	No change	15	Hoban et al. (2016); Champagne-Jorgensen et al. (2020); Kayyal et al. (2020); Leigh et al. (2020); Zhao et al. (2020); Keogh et al. (2021); Ray et al. (2021); Li et al. (2022); Zhang et al. (2022); Sun et al. (2023)
Corticosterone (circulating)	Increase	5	Jang et al. (2018); Zhao et al. (2020); Ruiz-González et al. (2022); Sarkar et al. (2022); Sun et al. (2023)
	Decrease	2	Li et al. (2022); Zhang et al. (2022)
	No change	6	Desbonnet et al. (2015); Hoban et al. (2016); Li et al. (2022); Yu et al. (2022)

### Meta-analysis of behavioral changes

**Figure 2:**
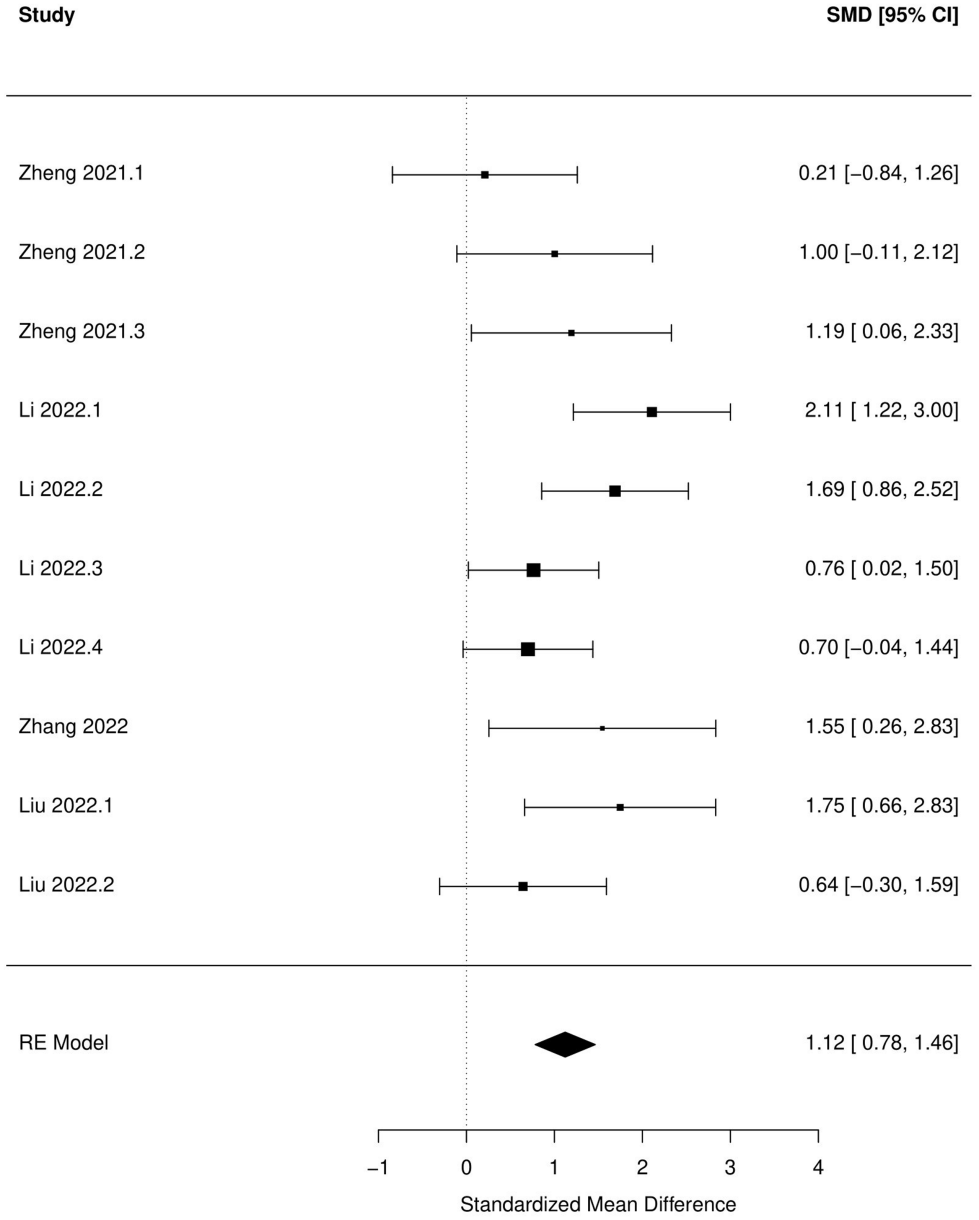
Forest plot of the random effects meta-analysis conducted using standardized mean differences of the latent time spend during Morris water maze test. “Study” is cross-referenced with the column “study population” in meta-analysis sheet in Supplementary material 2. Meta-data for each study can be assessed from this file. “SMD,” standardized mean difference; “RE,” random-effects model.

**Figure 3:**
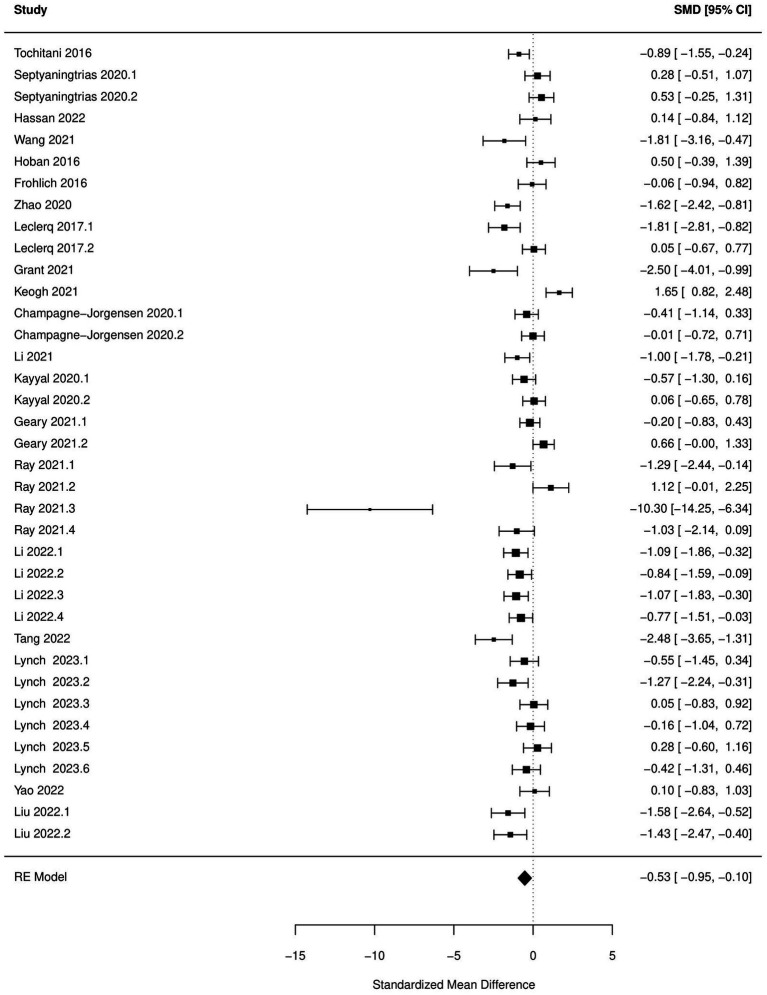
Forest plot of the random effects meta-analysis conducted using standardized mean differences of the time spend in center during open field test. “Study” is cross-referenced with the column “study population” in meta-analysis sheet in Supplementary material 2. Meta-data for each study can be assessed from this file. “SMD,” standardized mean difference; “RE,” random-effects model.

**Figure 4:**
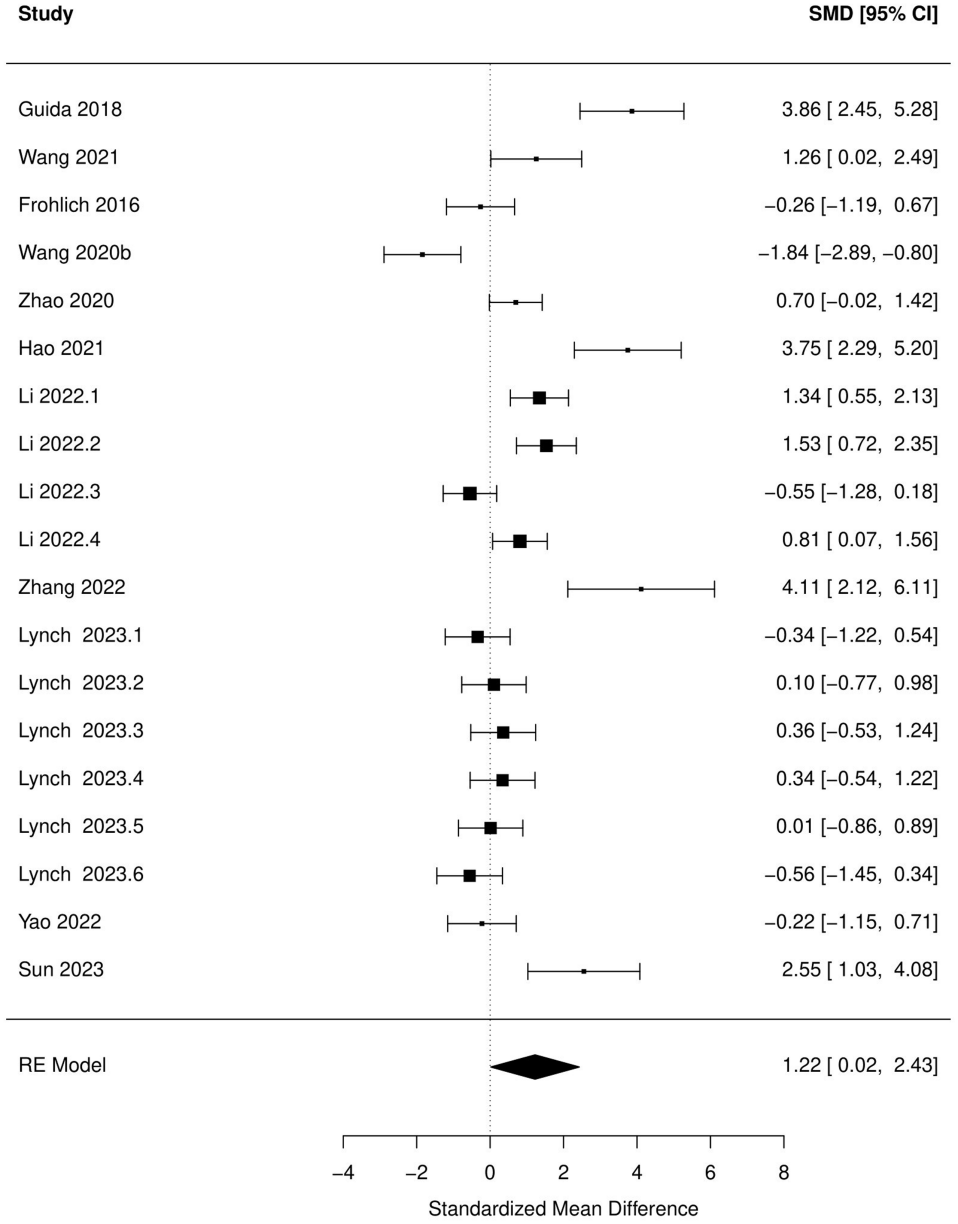
Forest plot of the random effects meta-analysis conducted using standardized mean differences of the immobile time during tail suspension test. “Study” is cross-referenced with the column “study population” in meta-analysis sheet in Supplementary material 2. Meta-data for each study can be assessed from this file. “SMD,” standardized mean difference; “RE,” random-effects model.

**Figure 5:**
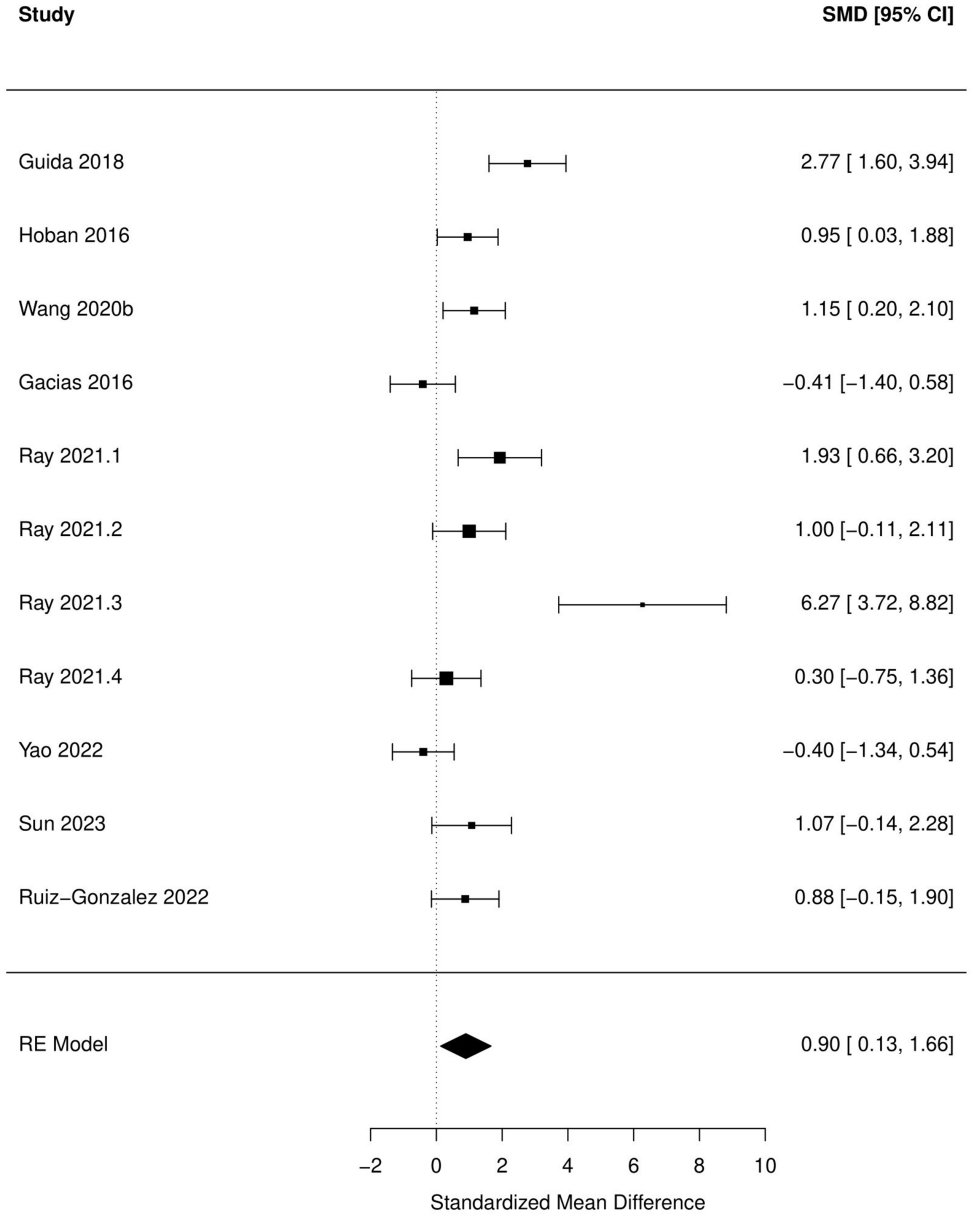
Forest plot of the random effects meta-analysis conducted on standardized mean differences of the immobile time during forced swim test. “Study” is cross-referenced with the column “study population” in meta-analysis sheet in Supplementary material 2. Meta-data for each study can be assessed from this file. “SMD,” standardized mean difference; “RE,” random-effects model.

Decrease in time spent in center (OFT), increased immobility time during FST and TST and increased escape latency (MWM) were significantly associated with antibiotic administration (*p* < 0.05; Figures 2–5; Table 8). Results of other tests were not associated with antibiotic consumption, but there was a trend (*p* < 0.10) toward statistical significance in case of time spent in open arm (EPM), and discrimination index (NOR). Sensitivity analysis was conducted by removing outliers but the results of OFT, FST, and TST remained statistically significant. Meta-analysis on the results of male mice administered orally revealed that time in center (OFT), immobility time (TST), and escape latency (MWM) remained significantly associated with changes in behavior, but the results of FST were not significant.

**Table 8:** Brief results of the meta-analysis conducted for some common behaviors tested in rodents administered antibiotics.

	Model A			Model B		
Behavioral phenotype	*N*	Pooled SMD (95% CI)	*p* value	*N*	Pooled SMD (95% CI)	*p* value
EPM-open arm time	22	−0.96 (−1.96, 0.03)	0.06	14	−1.08 (−2.44, 0.28)	0.11
FST-immobile time	11	0.90 (0.13, 1.66)	0.03	9	0.90 (−0.20, 1.99)	0.10
L/D-time in light	10	−0.28 (−2.59, 2.03)	0.79	8	−0.63 (−3.43, 2.16)	0.61
MWM-time in target quadrant	9	−2.14 (−4.88, 0.59)	0.11	5	−4.07 (−10.6, 2.47)	0.16
MWM-escape latency	10	1.12 (0.78, 1.46)	<0.01	8	1.12 (0.72, 1.52)	<0.01
NOR-discrimination index	17	−1.07 (−2.23, 0.10)	0.07	11	−1.02 (−2.22, 0.18)	0.09
OFT-time center	37	−0.53 (−0.95, −0.10)	<0.01	22	−0.52 (−0.92, −0.13)	0.01
TST-immobile time	19	1.22 (0.02, 2.43)	0.05	16	1.23 (0.02, 2.45)	0.05

Model A used data from all the study populations and Model B used data from populations with homogenous characteristics (male mice administered antibiotics orally). “N,” number of study populations; “SMD,” standardized mean difference calculated from random-effects meta-analytic model; and “CI,” confidence interval.

Based on subgroup analysis, number of antibiotics (single vs. multiple), age (pre-natal exposure and early infant vs. adolescent-adult), days of antibiotic administration (14 days vs. more than 14  days), and time between antibiotic administration and behavioral analysis (none vs. several days) did not influence associations between changes in behavior during OFT, FST, and TST and antibiotic consumption. Significant between-study heterogeneity (*p* < 0.05) was reported in all the meta-analytic models. There was a significant publication bias (*p* < 0.05) in the meta-analysis of all the behaviors tested, with the exception of escape latency in MWM.

## Discussion

We conducted a detailed systematic review to compile the results from articles that have described associations between antibiotic administration, gut microbiome changes, and changes in cognition, emotion, and behavior in rats and mice. This review highlights the uncertainties and inconsistencies in the current state of knowledge regarding aspects of the gut-brain-behavior axis, especially in the context of antibiotic-induced dysbiosis. Despite the large variation in behavioral tests applied, antibiotics used, bacterial taxa studied, and other factors, we were still able to identify some key patterns.

Significant increase in anxiety-like (OFT), depression-like behavior (FST and TST), and decrease in spatial cognition (MWM) after antibiotic use were the most outstanding results of this meta-analysis. These results should aid in strengthening the hypothesis that antibiotic-induced dysbiosis can manifest as cognitive and behavioral disturbances in rodents. Meta-analysis of the results of other behavioral tests, although not significant, also favored the hypothesis that antibiotic use can have deleterious effects on cognitive and behavioral functions in rodents. The discrepancy between the results of narrative review and meta-analysis can be explained by the likelihood that the studies were under-powered and there was a high chance of committing type II errors (Van Voorhis and Morgan, 2007), leading to the experimenters assigning a lack of statistical significance to a study despite the effect size and direction favoring the hypothesis. Inadequate sampling size in rodent studies has been acknowledged as one of the reasons why preclinical research often does not translate to human clinical applications (Bracken, 2009). One of the benefits of conducting a meta-analysis is the increase in the power to detect effects across studies (Der Simonian and Laird, 2015). The results of our meta-analysis additionally serve as an example of how meta-analysis of preclinical animal studies can aid in improving the power to detect effects in rodent studies. None of the studies provided a sample size justification for the number of animals used in these experiments. Considering that there is a preponderance of articles published on this topic, sample sizes should be calibrated to decrease the chance of committing type II errors.

Although these results are promising, caution should be exercised while interpreting these results. A significant publication bias indicates that results were more likely to be published if they were significant. This is consistent with the results of other systematic reviews published on rodent studies (Bonapersona et al., 2019; Wang et al., 2020; Theofilis et al., 2022). Under-representation of null or negative results can severely affect the overall conclusions and generalizability of any findings presented in this article.

There was a significant statistical between-study heterogeneity in the case of all except one behavioral test, which indicates that the results might not be generalizable. Multiple factors such as rodent species, strain, age, sex, antibiotics used, route of administration, and the duration of treatment could have played a role in this heterogeneity. However, the results of the meta-analysis still hold true after conducting analysis only on data collected from populations with a more homogenous background (i.e., only male mice administered oral antibiotics; Table 8). This reaffirms the evidence that antibiotic mediated gut microbiome changes can indeed cause cognitive and behavioral changes despite the differences in study designs and populations. Finally, several studies did not report whether investigators were blinded during different phases of the experiments. Several systematic reviews have found that the intervention effect is overestimated if investigators are not blinded during the conduct of experiment and assessment of outcomes (Karp et al., 2022). The correlation between increase in Phylum *Proteobacteria* in the gut after antibiotic intake and an increase in anxiety was the most significant association between gut bacteria and behavioral changes. Jeong et al. (2019) reported that a high-fat diet can potentially cause an increase in anxiety in mice by altering gut microbiota, especially by increasing relative abundance of *Proteobacteria* (Jeong et al., 2019). Simpson et al. (2020) conducted a systematic review of human and animal trials and found an association between inflammatory bowel disease, high gut levels of *Proteobacteria*, and anxiety/depression (Simpson et al., 2020). Hence, the results of our review are in line with existing scientific literature. Lipopolysaccharides secreted by *Proteobacteria* are known to be highly pro-inflammatory (Lin et al., 2020) and have been associated with pathogenesis of anxiety (Depino, 2015; van Eeden et al., 2021).

Despite the above results, we were not able to identify study designs and populations that can elicit behavioral changes post-antibiotic administration consistently. The following factors might have played a role in this lack of consistency:

### Study subjects

Rats and mice have inherently different characteristics that can alter their responses to specific stimuli. For example, rats are more habituated to human handling as compared to mice and this can reduce certain erratic behaviors during the conduct of anxiety tests (Wang et al., 2020). Rats and mice diverged around 15–20 million years ago and from an evolutionary perspective, they differ in serotonergic innervation and levels of neurogenesis (Ellenbroek and Youn, 2016). Rats and mice exhibit different behaviors during swimming leading to discrepancies in read-outs of tests such as MWM (Othman et al., 2022).

Differences in performances during behavioral paradigms have also been observed at a strain level. For example, van Gaalen and Steckler (2000) compared the differences in locomotion and anxiety-like behavior using L/D box and EPM, and found significant differences in the performances of four different strains of mice. C57BL mice consistently develop spatial memory in MWM, whereas BALB mice have been reported to perform poorly by some scientists (Van Voorhis and Morgan, 2007). Even the same strains of rats obtained from different vendors have been reported to differ in anxiety levels based on FST (Porsolt et al., 1978; Bogdanova et al., 2013). Age and sex also play a role in the behavioral phenotypes observed during FST (Bogdanova et al., 2013), OFT (Seliger, 1977; Börchers et al., 2022), EPM (Börchers et al., 2022), and NOR (Sutcliffe et al., 2007).

Results generated from rodent models should only be generalized to human populations after exercising great caution. We also considered studies from other animal models such as non-human primates, and humans at the inception of this review. However, we were not able to find any studies that involved non-human primates and were within the scope of this review. Non-human primates are evolutionarily closer to humans than rodents and are the best models to study psychiatric symptoms and behavioral paradigms. These models can be used to study the impact of antibiotic-induced gut dysbiosis on complex human behaviors and psychiatric disorders such as addiction, sociability, response to stress, etc. Studies similar to those included in this review can be conducted on non-human primates in order to generate further pre-clinical evidence that antibiotics can indeed lead to behavioral changes by altering the gut microbiome.

### Antibiotics

The choice and combination of antibiotics, route of administration, and duration between antibiotic administration and behavioral testing can all play a major role in determining the behavioral outcomes in rodents. Ampicillin, vancomycin, and penicillin V were the three most common antibiotics that were administered individually. Ampicillin is a broad-spectrum antibiotic and effective against both Gram-positive and-negative bacteria, whereas vancomycin and penicillin V are narrow spectrum antibiotics efficacious against only Gram-positive bacteria (Kahn and Line, 2010). Using antibiotics with differing modes of action and spectrum of action leads to differences in the gut dysbiosis induced (Septyaningtrias et al., 2020; Liu et al., 2022). It should also be noted that not all antibiotics have a potentially depressogenic effect. For example, minocycline (a broad-spectrum antibiotic) was used in one of the experiments to test its anxiolytic effect (Yao et al., 2022).

It can be hypothesized that using a cocktail of antibiotic classes can induce a broader disruption of the gut microbiome as compared to a single antibiotic. In the studies included in this review, a combination of ampicillin, neomycin, or vancomycin with other antibiotics was the most common cocktail used and should have hypothetically induced a greater disruption and hence, bigger changes in behaviors recorded. Surprisingly, this was not the case based on the narrative review and subgroup analysis. On the flip side, testing a single antibiotic for behavioral changes might have far more direct translational potential for clinical practice because single antibiotics are more commonly prescribed in clinical practice and a combination of antibiotics are reserved for more complicated cases (Leekha et al., 2011).

Some of the antibiotics used in these studies, such as metronidazole, ciprofloxacin, clindamycin etc. are easily absorbable from intestines (Hackam et al., 1998; Kasten, 1999). Systematic effects of these antibiotics after oral absorption can potentially confound the true relation between gut dysbiosis and behavioral alterations, especially if they cross blood–brain barrier. For example, metronidazole can easily cross the blood–brain barrier (Tsai and Chen, 2003; Nau et al., 2010) and have been known to occasionally cause neuropathy and encephalopathy in humans (Sørensen et al., 2020). Ciprofloxacin has also been known to be directly toxic to the central nervous system (Ilgin et al., 2015).

Route of administration and time between antibiotic administration and evaluation of gut microbial populations are other factors that can bias the interpretation of the studies included in this review. Antibiotics given orally are far more likely to cause disruptions in gut bacterial populations as compared to parenteral route (Kelly et al., 2020; Zhou et al., 2020). In a very limited number of studies included in this review, rodents administered antibiotics parenterally displayed less changes in behavior and gut dysbiosis as compared to rodents given antibiotic orally. The impact of antibiotics on the disruption of gut microbiome is usually reversible, although the time taken to full recovery is still a question of research. Ray et al. (2021) demonstrated how the gut microbiome gradually returned to near normalcy within 60 days of vancomycin treatment in BALB/C mice. This was accompanied by a return to normal behavior. Baseline structure of gut microbial populations can also influence the response to antibiotics (Zhou et al., 2020). Unfortunately, such baseline data were missing from a vast majority of the studies.

### Test setup

We did not include the reported testing methodology in our risk-of-bias assessment because each test was not conducted in every study. The variability in how the tests were conducted might be one of the biggest factors influencing the results of behavioral outcomes. The detailed testing methodology was often not provided by the authors. Even when reported, there were huge discrepancies in testing methodology. For example, the trial time in OFT ranged from 3 min (Arslanova et al., 2021) to 2 h (Tochitani et al., 2016). This can have a significant effect on the exploratory behavior because the tendency to explore might decrease over time (Tochitani et al., 2016). Similarly, Septyaningtrias et al. (2020) and Hassan et al. (2022) considered the central 60 and 72% of the arena as the “central zone” in OFT, respectively. The designation of central area can have a direct impact on evaluation of the time spent in center, which is the main OFT metric used by several studies (Tochitani et al., 2016). The time between acquisition phase and testing phase (inter-trial period) also varied from 1 h (Fröhlich et al., 2016; Hoban et al., 2016; Lach et al., 2020) to 1 day (Saunders et al., 2020) across the studies. Sutcliffe et al. (2007) have demonstrated that the ability of male rats to distinguish between familiar and novel objects as well as overall object exploration time decreases drastically after 1 h of inter-trial period. We have highlighted only a few discrepancies that were reported in these studies. There are a huge range of factors that can influence the results of behavioral tests including dimensions of the apparatus, ambient temperature and humidity, ambient light, human handling, and habituation to a procedure (Rodgers and Dalvi, 1997; Kaidanovich-Beilin et al., 2011; Antunes and Biala, 2012; Bogdanova et al., 2013; Seibenhener and Wooten, 2015). We realize that there are no standardized experimental protocols to conduct these behavioral tests that have been uniformly adopted by the scientific community. But the authors can still provide details on the testing methodology in the supplementary material so that the behavioral outcomes can be compared across the studies.

### Analysis of the gut microbiome

Although nearly all of the studies used 16S rRNA sequencing as a tool to study gut microbiome, the methodologies used to generate, process and analyze the sequencing data varied widely. Varying methodological considerations include the target regions sequenced, bioinformatic pipelines used, databases used for classification of bacterial taxa and statistical methods used. Comparison of performances between commonly used pipelines have revealed significant differences in the structure of bacterial populations and these differences were further dependent on the gene databases used (Almeida et al., 2018; López-García et al., 2018). Statistical methods, such as ANCOM-BC, LefSe, and Aldex2 used to identify differentially abundant taxa across experimental groups can produce discordant results to varying degrees (Wallen, 2021; Nearing et al., 2022). These are some of the challenges in generalizing the results of microbiome studies and are not limited to articles included here. Moreover, the results of 16S rRNA and shotgun sequencing can yield tens to hundreds of different bacterial taxa and it is not feasible for scientists to report on each bacterial taxon detected. However, authors should at the very least share the taxa tables generated by the bioinformatic pipelines, share the codes used using a public repository, and upload the raw reads on a gene repository (such as GenBank). This way adequate data will be available for the scientific community to compare the results across different studies. Direct correlations between behavior and abundances of bacterial taxa were seldomly reported in these studies. Availability of genomic data might have also allowed for an estimation of behavioral changes and bacterial taxa using a meta-analytic approach. Finally, differences in sampling and storage procedures can further contribute to the heterogeneity of the results.

## Future direction—research domain criteria approach

To provide a better framework for conduction of psychiatric research, National Institute of Mental Health (NIMH) launched the Research Domain Criteria (RDoC) in 2013 (Cuthbert and Insel, 2013). The RDoC agenda strives to explicate fundamental bio-behavioral dimensions that span multiple current heterogeneous disorder categories (Insel et al., 2010). Since the RDoC domains are mainly constructed by basic neuroscience work including animal models, the animal model studies will have a much better opportunity to be aligned well with the RDoC frame. This can potentially lead to addressing fundamental questions on establishing neurobiological models for mental health disorders (Anderzhanova et al., 2017). In this regard, future studies of microbiota-gut-brain axis using animal models may seriously consider implementing the RDoC approach, by focusing on dimensional psychopathologies and underlying neurobiological mechanisms, avoiding focusing on categorical psychiatric diagnoses. In this regard, commonly used animal experiments to elicit behavioral phenotypes, can be considered within the RDoC framework as well (Table 2), which should be carefully considered when designing studies of microbiota-gut-brain axis.

## Limitations

This study was limited by the various methodological variations, biases, incomplete reporting, and heterogeneity of the rodent populations studied. We limited search to the English language and did not search gray literature such as dissertations or preprint repositories, which might have led to the exclusion of some studies. We did not conduct a meta-analysis of the molecular mechanisms because these were not part of the original search string. Some of the terms such as anxiety or depression might not have direct translational value and this topic is debated among neuroscientists (Molendijk and de Kloet, 2019). However, we used these terms because of their simple interpretations and these are widely used in the existing literature, including the studies we collected the data from. We combined the results of different tests to form a behavioral phenotype. These tests might evaluate the same behavioral phenotype under different conditions and the results might differ slightly (Steimer, 2011). This was done to simplify the narrative review and we have provided details for individual tests in the Supplementary material 2. We would also like to reiterate that significant heterogeneity (due to confounding factors such as sex, strain, age, antibiotics used, and different husbandry between studies and methodological variations), publication bias, small sample sizes, and inadequate data reporting limits the potential generalizability of the results of the meta-analysis.

## Conclusion

The results of this systematic review meta-analysis provide some evidence that antibiotic-induced gut dysbiosis can indeed cause some behavioral changes in rodents. However, the heterogeneity between study populations, behavioral testing and microbiome evaluation makes it difficult to generalize these results. We are still far from establishing a model of antibiotic-induced gut dysbiosis and behavioral change in rodent models that can consistently reproduce the same results.

## Data availability statement

The original contributions presented in the study are included in the article/Supplementary material, further inquiries can be directed to the corresponding author.

## Author contributions

SHa: conceptualization, conducting review, data analysis, and writing. SHw: writing. JC: conceptualization and writing. All authors contributed to the article and approved the submitted version.

## Funding

This research was supported by startup funds provided by the University of Nebraska at Omaha and the Nebraska Food for Health Center to JC. Research reported in this publication was supported by the Office of the Director, National Institutes of Health of the National Institutes of Health under Award Number K01OD030514 awarded to JC. The content is solely the responsibility of the authors and does not necessarily represent the official views of the National Institutes of Health.

## Conflict of interest

The authors declare that the research was conducted in the absence of any commercial or financial relationships that could be construed as a potential conflict of interest.

## Publisher’s note

All claims expressed in this article are solely those of the authors and do not necessarily represent those of their affiliated organizations, or those of the publisher, the editors and the reviewers. Any product that may be evaluated in this article, or claim that may be made by its manufacturer, is not guaranteed or endorsed by the publisher.
