# Supplementary material for: Antibiotic-induced gut dysbiosis and cognitive, emotional, and behavioral changes in rodents: a systematic review and meta-analysis
Source: Front Neurosci. 2023 Sep 1;17:1237177. doi: 10.3389/fnins.2023.1237177 (PMC10504664; doi:10.3389/fnins.2023.1237177)
Supplement: Supplementary file 1 [file Table_1.docx]

Search strings

Web of Science and Scopus:

(antibiotic* OR antimicrobial* OR anti-biotic* OR anti-microbial* OR antibacterial* OR anti-bacterial*) AND

(microbiom* OR metagenom* OR  16s*  OR  microbiot*) AND

(autis* OR bipolar OR attent* OR schizo* OR ASD OR dement* OR frailty OR Alzheimer* OR affective disorder* OR Parkinson* OR depress* OR neurodegen* OR mood* OR stress* OR psych* OR depress* OR anxi* OR anorexia OR cogni* OR neuro* OR behav* OR mental* OR soci* OR pain* OR nocicep* OR memory* OR spati* OR fear*) AND

(mice OR rat OR rats OR rodent* OR mouse)

PUBMED:

("Microbiota" [MESH] OR "microbio*" [tiab] OR "metagen*" [tiab] OR "16s" [tiab]) AND ("Anti-Infective Agents"[Mesh] OR "anti-bacterial" [tiab] OR "anti-infective" [tiab] OR "antibacterial" [tiab] OR "antiinfective" [tiab] OR "anti-microbial" [tiab] OR "antimicrobial" [tiab] OR "antibiotic" [tiab] OR "antitubercular" [tiab] OR "penicillin" [tiab] OR "amoxicillin" [tiab] OR "carbapenem" [tiab] OR "cephalosporin" [tiab] OR "macrolide" [tiab] OR "quinolone" [tiab] OR "glycopeptide" [tiab] OR "aminoglycoside" [tiab] OR "tetracycline" [tiab] OR "tigecycline" [tiab] OR "daptomycin" [tiab] OR "streptogramin" [tiab] OR "colistin" [tiab] OR "linelozid" [tiab] OR "trimethoprim" [tiab] OR "sulphonamide" [tiab] OR "nitrofurantoin" [tiab] OR "Fosfomycin" [tiab] OR "amikacin" [tiab] OR "ampicillin" [tiab] OR "benzylpenicillin" [tiab] OR "cefalexin" [tiab] OR "cephalexin" [tiab] OR "cefazolin" [tiab] OR "cephazolin" [tiab] OR "chloramphenicol" [tiab] OR "clindamycin" [tiab] OR "cloxacillin" [tiab] OR "doxycycline" [tiab] OR "gentamicin" [tiab] OR "metronidazole" [tiab] OR "spectinomycin" [tiab] OR "sulphamethoxazole" [tiab] OR "sulfamethoxazole" [tiab] OR "azithromycin" [tiab] OR "cefixime" [tiab] OR "cephixime" [tiab] OR "cefotaxime" [tiab] OR "cephotaxime" [tiab] OR "ceftriaxone" [tiab] OR "cephtriaxone" [tiab] OR "cefuroxime" [tiab] OR "cephuroxime" [tiab] OR "ciprofloxacin" [tiab] OR "clarithromycin" [tiab] OR "piperacillin" [tiab] OR "vancomycin" [tiab] OR "ceftazidime" [tiab] OR "cephtazidime" [tiab] OR "meropenem" [tiab] OR "polymyxin" [tiab] OR "clofazimine" [tiab] OR "dapsone" [tiab] OR "rifampicin" [tiab]) AND ("Psychiatry and Psychology Category" [MESH] OR "autis*" [tiab] OR "bipolar" [tiab] OR "attent*" [tiab] OR "schizo*" [tiab] OR "ASD" [tiab] OR "dement*" [tiab] OR "frailty" [tiab] OR "Alzheimer*" [tiab] OR "affective disorder" [tiab] OR "Parkinson*" [tiab] OR "depress*" [tiab] OR "neurodegen*" [tiab] OR "mood*" [tiab] OR "stress*" [tiab] OR "psych*" [tiab] OR "anxi*" [tiab] OR "anorexia" [tiab] OR "cogni*" [tiab] OR "neuro*" [tiab] OR "behav*" [tiab] OR "mental*" [tiab] OR "soci*"[tiab] OR "pain" [tiab] OR "nocicep*" [tiab] OR "memory" [tiab] OR "spati*" [tiab] OR "mental health" [tiab] OR "fear" [tiab]) AND ("mice" [tiab] OR "rat*" [tiab] OR “mouse” [tiab] OR “rodent*” [tiab])
